# Association Between Obesity, Digital Screen Time, and Early-Onset Hypertension in Adolescents: A Prospective Cohort Study

**DOI:** 10.7759/cureus.79975

**Published:** 2025-03-03

**Authors:** Sadaf Yasin, Muhammad Hasnain, Fahad R Khan, Kamran Aslam, Nasir Farooq, Atif Ihsan

**Affiliations:** 1 Internal Medicine, Shaheed Mohtarma Benazir Bhutto Institute of Trauma, Larkana, PAK; 2 Cardiology, Lady Reading Hospital, Peshawar, PAK

**Keywords:** adolescents, blood pressure regulation, digital screen time, hypertension, obesity, physical activity, public health interventions, sedentary lifestyle

## Abstract

Objective

This study aimed to assess the associations between obesity, digital screen time, and early-onset hypertension in adolescents. Specifically, it examined the independent and combined effects of these factors on blood pressure (BP) regulation.

Methods

A prospective cohort study was conducted among 350 adolescents (N = 350) aged 10-18 years, with an equal distribution of obese (n = 175, 50.0%) and non-obese (n = 175, 50.0%) participants. Digital screen time was categorized into recreational and educational use, with excessive screen time defined as >3 hours/day. BP was measured three times per participant using a validated oscillometric device, following the American Academy of Pediatrics (AAP) guidelines. Hypertension was defined as BP ≥ 95th percentile for age, sex, and height. Multivariate logistic regression was employed to assess the association between obesity, screen time, and hypertension, adjusting for physical activity, socioeconomic status (SES), family history of hypertension, and dietary factors.

Results

A total of 82 participants (23.4%) were hypertensive, with a significantly higher prevalence in obese adolescents (n = 64, 36.6%) compared to non-obese adolescents (n = 18, 10.3%) (p < 0.001). Excessive screen time (>3 hours/day) was an independent predictor of hypertension (adjusted odds ratio (aOR) = 2.58, 95% CI: 1.73-3.86; p < 0.001). Recreational screen time (>3 hours/day) was more strongly associated with hypertension (aOR = 3.12, 95% CI: 2.10-4.02) compared to educational screen time (aOR = 1.75, 95% CI: 1.21-2.44).

Adolescents with both obesity and excessive screen time had the highest hypertension prevalence (52.4%), significantly exceeding those with either risk factor alone. The combined risk group had an aOR = 5.14 (95% CI: 3.28-7.56), suggesting a potential interaction effect, though formal mediation analysis was not performed. Low physical activity (Physical Activity Questionnaire for Older Children (PAQ-C) score < 2.33) was also associated with hypertension (aOR = 1.62, 95% CI: 1.04-2.51; p = 0.033).

Conclusion

Obesity and excessive screen time are independently and jointly associated with early-onset hypertension in adolescents. These findings emphasize the need for targeted interventions, including routine BP screening for high-risk adolescents, school-based digital wellness programs, and structured physical activity initiatives.

While this study establishes associations, not causality, further longitudinal research is required to explore the causal pathways and potential interaction effects between obesity, screen time, and hypertension.

## Introduction

Hypertension in adolescents is an escalating public health concern, intricately linked to the rising prevalence of obesity and increased digital screen time among youth. Recent studies have indicated that approximately 14% of children and adolescents worldwide exhibit elevated or high blood pressure (BP), with prevalence rates varying significantly across different regions, such as South Asia and sub-Saharan Africa. In some urban populations, adolescent hypertension prevalence has been reported to reach as high as 22%, underscoring the global nature of the issue and the need for international public health strategies to mitigate its impact. In the United States, approximately 14% of children and adolescents exhibit elevated BP, with higher rates observed among obese individuals [1]. Obesity, which affects a significant proportion of the pediatric population, is a well-established risk factor for the development of hypertension in this age group [2].

The pathophysiology of hypertension in adolescents with obesity involves complex interactions between genetic, environmental, and behavioral factors. Having too much fat makes insulin less effective, the sympathetic nervous system is overactive, and changes in the structure and function of blood vessels are all major causes of high BP [3]. Concurrently, sedentary behaviors, particularly prolonged digital screen time, have been associated with unfavorable cardiovascular profiles, including increased BP and adiposity [4]. Beyond sedentary behavior, prolonged digital screen exposure contributes to hypertension risk through multiple physiological mechanisms. First, prolonged use of screens has been linked to issues with autonomic function and higher cortisol levels. This is because of the prolonged use of video games, social media, and digital content, which causes vascular resistance and long-term high BP. Second, blue light exposure from digital devices, particularly in the evening, suppresses melatonin secretion, causing sleep disturbances and disrupted circadian rhythms. These disruptions increase sympathetic nervous system activity and reduce parasympathetic tone, further exacerbating BP dysregulation. Third, long-term screen time is connected to higher levels of inflammatory markers like interleukin (IL)-6 and tumor necrosis factor-alpha (TNF-α), which cause vascular inflammation, endothelial dysfunction, and high BP.

Current management strategies for adolescent hypertension emphasize lifestyle modifications as the cornerstone of therapy. These include dietary interventions, such as the adoption of the Dietary Approaches to Stop Hypertension (DASH) diet, regular physical activity, and weight reduction in overweight or obese individuals [5]. Pharmacological treatment is generally reserved for cases in which lifestyle changes are insufficient or when hypertension is severe or accompanied by target-organ damage [6].

Despite these recommendations, there is a paucity of data specifically addressing the interplay between obesity, digital screen time, and early-onset hypertension in adolescents. Understanding this relationship is crucial for developing targeted interventions to mitigate the burden of cardiovascular disease later in life. While this study examines the relationship between obesity, screen time, and hypertension in adolescents, it is important to note that our findings describe associations rather than causality. Given the observational nature of our research, we cannot infer direct cause-and-effect relationships between these variables. Further longitudinal and interventional studies are required to determine the causal pathways underlying these associations.

This prospective cohort study aimed to fill this gap by investigating the association between obesity, digital screen time, and early-onset hypertension in adolescents. Existing studies have primarily focused on limited geographic populations or single-center analyses, often lacking robust sample sizes and longitudinal follow-ups. Additionally, previous research has not adequately controlled for confounders, such as socioeconomic status (SES), dietary patterns, and genetic predispositions [7]. Addressing these limitations, our study aimed to provide a more comprehensive and generalizable assessment of the risk factors contributing to hypertension in adolescents.

The study's results could change clinical practice by revealing risk factors that can be changed and that lead to high BP in teens. This will help make better plans for prevention and treatment. Early identification and intervention are paramount, given that hypertension during adolescence is a predictor of cardiovascular morbidity and mortality in adulthood.

## Materials and methods

Author coordination and logistics

This study was conducted as a collaborative effort between researchers from the Shaheed Mohtarma Benazir Bhutto Institute of Trauma and the Emergency Response Center, Larkana, Pakistan, and Lady Reading Hospital, Peshawar, Pakistan. All the authors were actively involved in the study design, data collection, and statistical analysis, ensuring a cohesive and well-coordinated research process.

To maintain seamless coordination between the two centers, biweekly virtual meetings were conducted, supplemented by periodic in-person visits. A shared cloud-based repository was used for protocol standardization, data security, and manuscript development, allowing for real-time collaboration. Each institution was designated a lead investigator responsible for overseeing participant recruitment, quality control, and adherence to ethical standards at their respective centers.

Study design and setting

A prospective cohort study was conducted from January 1, 2023, to December 31, 2023, at two tertiary healthcare centers in Pakistan: Shaheed Mohtarma Benazir Bhutto Institute of Trauma and Emergency Response Center and Lady Reading Hospital. This study aimed to investigate the association between obesity, digital screen time, and early-onset hypertension in adolescents. Participants were recruited from outpatient pediatric clinics (clinical sample) and community-based health screenings (general population sample) to ensure diverse representation. Urban, peri-urban, and rural areas were classified based on Pakistan Bureau of Statistics census data.

Study population and eligibility criteria

Adolescents aged 10-18 years who attended routine pediatric consultations or received referrals for cardiovascular risk assessment participated in this study. To be included in the study, participants needed documented height, weight, BMI percentile, self-reported or parental reports of digital screen time, and at least three separate BP measurements recorded during the study period. Hypertension diagnosis was defined using percentile cutoffs for age, sex, and height, based on the American Academy of Pediatrics (AAP) guidelines.

Exclusion criteria included adolescents with secondary hypertension due to renal, endocrine, or cardiovascular disorders; those using antihypertensive or corticosteroid medications; and those with chronic illnesses affecting cardiovascular health, including type 1 diabetes and congenital heart disease. BP was measured thrice per participant in a seated position using validated oscillometric devices. Home BP monitoring and ambulatory BP monitoring (ABPM) were not used because of feasibility constraints. Secondary hypertension was ruled out based on clinical assessment and medical records.

Sample size calculation

The sample size was determined based on epidemiological data on the prevalence of hypertension among obese adolescents in Pakistan. A meta-analysis published in the Archives of Public Health reported a pooled prevalence of 12.16% of hypertension among Pakistani adolescents [8]. Given these findings, an estimated prevalence of 12% was used for sample size calculation. Utilizing a 95% confidence interval and 80% power, the required sample size was calculated as 350 participants, divided equally into two groups: 175 obese and 175 non-obese adolescents. The 50:50 obese-to-non-obese ratio was intended to enhance statistical power and enable balanced comparisons. Statistical adjustments were applied to account for differences in the real-world obesity prevalence.

Data collection and variables

Obesity was classified based on the Centers for Disease Control and Prevention (CDC) BMI percentiles, with obesity defined as a BMI ≥ 95th percentile for age and sex [9].

Physical activity levels were assessed using the Physical Activity Questionnaire for Older Children (PAQ-C) (Appendix A), a validated seven-day recall instrument that evaluates habitual physical activity in school, extracurricular activities, and leisure settings [10]. PAQ-C was used instead of accelerometry owing to feasibility constraints. Each item was scored on a five-point Likert scale, and the final PAQ-C score was classified as low (<2.33), moderate (2.33-3.66), or high (>3.66). The PAQ-C has been validated against objective activity measures, such as accelerometry, and aligns with the World Health Organization (WHO) 2020 Guidelines on Physical Activity and Sedentary Behavior, which recommend at least 60 minutes of moderate-to-vigorous physical activity per day for children and adolescents [11].

Digital screen time was defined as all screen-based activities including television, gaming, smartphones, tablets, laptops, and desktops. Screen time was categorized into recreational (e.g., gaming, social media, entertainment) and educational (e.g., online learning, homework) use to determine its differential impact on hypertension risk. The screen time questionnaire, adapted from Vizcaino et al. [12], classifies exposure as low (<2 hours/day), moderate (2-4 hours/day), or high (>4 hours/day) (Appendix B).

Dietary intake was evaluated using a semi-quantitative Food Frequency Questionnaire (FFQ) (Appendix C) that was adapted to culturally relevant dietary patterns in Pakistan by the authors. To enhance external validity, the FFQ score classification was based on previous literature rather than being derived from the study sample. The FFQ categorizes dietary habits into three adherence levels: high (≥75th percentile, high fruit/vegetable intake, and low fast-food consumption), moderate (50th-74th percentile), and low (<50th percentile, frequent consumption of processed foods and sugar-sweetened beverages). Parental verification was sought in younger participants to minimize recall bias. The original version of the FFQ has been widely used in nutritional epidemiology [13], and the semi-quantitative version has been validated in South Asian populations [14].

Statistical analysis

Descriptive statistics were computed using IBM SPSS Statistics for Windows, Version 26 (Released 2019; IBM Corp., Armonk, New York, United States) and Python (SciPy and StatsModels libraries). Continuous variables were analyzed using independent t-tests, and categorical variables were assessed using the chi-square test. Multivariate logistic regression models were employed to assess associations, adjusting for key confounders, including age, sex, BMI percentile, SES, dietary habits (FFQ score), urban-rural classification, and family history of hypertension. Wald test statistics confirmed the significance of the key predictors, including obesity, screen time, and low physical activity. Likelihood ratio tests (LRTs) validated model improvement when additional confounders were included.

Ethical considerations

Ethical approval was obtained from the Shaheed Mohtarma Benazir Bhutto Institute of Trauma and Emergency Response Center, Larkana (Approval Reference: SMBB/ERC-2022/091), and the Lady Reading Hospital, Peshawar (Approval Reference: 563/IRB/LRH/MTI). Written informed consent was obtained from parents or legal guardians, and adolescent participants provided consent. Data confidentiality was ensured through secure, de-identified electronic records accessible only to authorized research personnel.

## Results

A total of 350 adolescents (N = 350) participated in the study, with 175 (50.0%) classified as obese and 175 (50.0%) as non-obese. The mean age of participants was 14.6 ± 2.3 years, with a nearly equal gender distribution (178 males (50.9%) and 172 females (49.1%)).

Obese participants had a significantly higher median BMI of 31.2 kg/m² (IQR: 29.8-33.5 kg/m²) compared to their non-obese counterparts, who had a median BMI of 21.4 kg/m² (IQR: 19.6-22.7 kg/m²) (p < 0.001). Socioeconomic differences were also observed, with a higher proportion of obese adolescents residing in urban areas (127 (72.6%)) compared to non-obese adolescents (89 (50.9%)) (p < 0.001).

A positive family history of hypertension was reported more frequently in the obese group (87 (49.7%)) than in the non-obese group (32 (18.3%)) (p < 0.001). Lifestyle factors also differed significantly between groups: prolonged screen time (>3 hours/day) was reported by 143 (81.7%) obese participants compared to 58 (33.1%) non-obese participants (p < 0.001), and fewer obese adolescents met recommended daily physical activity levels (<60 minutes/day in 121 (69.1%)) obese adolescents vs. 47 (26.9%) non-obese adolescents; p < 0.001).

The baseline demographic and clinical characteristics of the study population, stratified by obesity status, are presented in Table 1. The table includes test statistics for age, gender, BMI, urban residence, family history of hypertension, screen time, and physical activity.

**Table 1:** Baseline demographic and clinical characteristics of the study population This table summarizes the demographic and clinical characteristics of the study population categorized by obesity status. Test statistics include t-values for independent t-tests, chi-square values (χ²) for categorical variables, and U-values for Mann-Whitney U tests where applicable. Asterisks (*) indicate statistical significance at p < 0.05.

Variable	Total (N = 350)	Obese (N = 175)	Non-obese (N = 175)	Test statistic	p-value
Age (years, mean ± SD)	14.6 ± 2.3	14.9 ± 2.2	14.3 ± 2.5	t = 2.14	0.032*
Male, N (%)	178 (50.9)	92 (52.6)	86 (49.1)	χ² = 0.42	0.518
Female, N (%)	172 (49.1)	83 (47.4)	89 (50.9)	χ² = 0.42	0.518
BMI (kg/m², median (IQR))	25.6 (21.4–31.2)	31.2 (29.8–33.5)	21.4 ([19.6–22.7)	U = 12.45	<0.001*
Urban residence, N (%)	216 (61.7)	127 (72.6)	89 (50.9)	χ² = 15.62	<0.001*
Family history of hypertension, N (%)	119 (34.0)	87 (49.7)	32 (18.3)	χ² = 27.38	<0.001*
Screen time > 3 hours/day, N (%)	201 (57.4)	143 (81.7)	58 (33.1)	χ² = 62.84	<0.001*
Physical activity < 60 minutes/day, N (%)	168 (48.0)	121 (69.1)	47 (26.9)	χ² = 51.26	<0.001*

**Figure 1:**
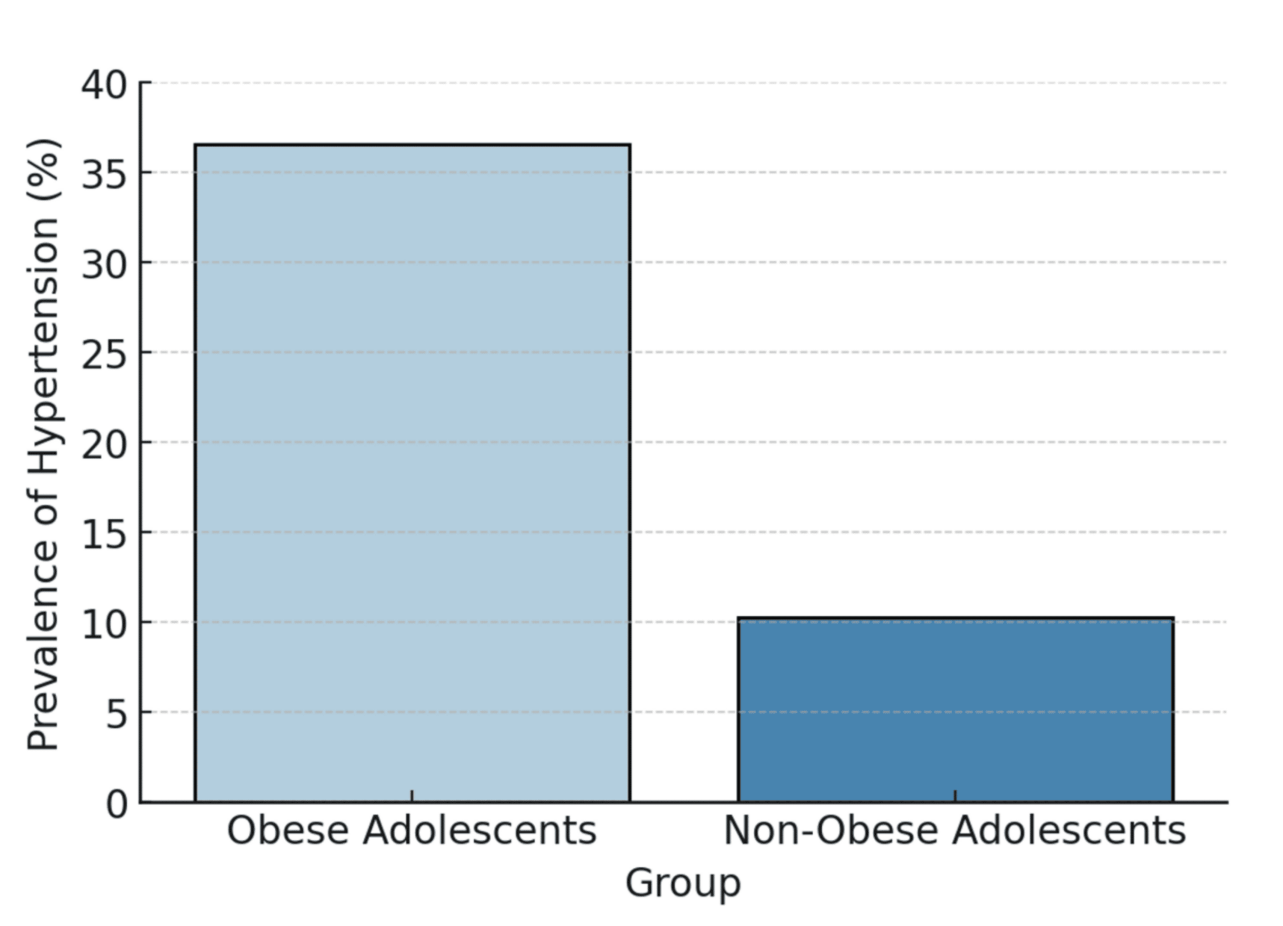
Prevalence of hypertension in obese versus non-obese adolescents

Among the overall sample, 82 participants (23.4%) were hypertensive, with a significantly higher prevalence in the obese group (64 (36.6%)) compared to the non-obese group (18 (10.3%)) (p < 0.001). Figure 1 illustrates the difference in hypertension.

When comparing hypertensive versus normotensive adolescents, significant differences emerged in BP (systolic blood pressure (SBP) and diastolic blood pressure (DBP)), screen time, physical activity, and dietary adherence, as shown in Table 2.

**Table 2:** Blood pressure and lifestyle factors in hypertensive versus normotensive adolescents This table presents the blood pressure parameters and associated lifestyle factors in hypertensive and normotensive adolescents. Independent samples t-tests were used to compare continuous variables (SBP and DBP), while categorical variables (screen time, PAQ-C, and FFQ scores) were analyzed using the chi-square test. The asterisk symbol (*) indicates statistical significance at p < 0.05. SBP: systolic blood pressure; DBP: diastolic blood pressure; PAQ-C: Physical Activity Questionnaire for Older Children; FFQ: Food Frequency Questionnaire

Variable	Hypertensive (N = 82)	Normotensive (N = 268)	p-value
SBP (mmHg, mean ± SD)	128.7 ± 10.6	116.4 ± 8.9	<0.001*
DBP (mmHg, mean ± SD)	82.1 ± 8.4	75.6 ± 6.7	<0.001*
Screen time > 3 hours/day, N (%)	68 (82.9)	133 (49.6)	<0.001*
PAQ-C score < 2.33, N (%)	53 (64.6)	115 (42.9)	<0.001*
FFQ score < 50th percentile, N (%)	47 (57.3)	99 (36.9)	<0.001*

Hypertensive adolescents had higher SBP and DBP, were more likely to report excessive screen time, engaged in lower levels of physical activity, and exhibited poorer dietary adherence compared to their normotensive counterparts.

The mean PAQ-C score was significantly lower among hypertensive adolescents (2.15 ± 0.48) compared to normotensive adolescents (3.02 ± 0.52, p < 0.01), indicating reduced physical activity in those with hypertension. Additionally, adolescents reporting more than four hours of total screen time per day had a significantly higher prevalence of hypertension (OR: 2.78, 95% CI: 1.95-3.84; p < 0.01). Dietary adherence, as measured by FFQ scores, was poorer in hypertensive adolescents (52nd percentile) compared to non-hypertensive participants (68th percentile) (p < 0.05).

Further stratification of screen time into recreational and educational use revealed that adolescents engaging in more than four hours per day of recreational screen activities (e.g., gaming, social media) had a significantly higher prevalence of hypertension (OR: 3.12, 95% CI: 2.10-4.02; p < 0.01). Those whose screen time was predominantly educational (e.g., online learning, homework) for more than four hours per day also showed an association with hypertension (OR: 1.75, 95% CI: 1.21-2.44; p = 0.03), although the effect was smaller compared to recreational screen time.

Table 3 outlines the standardized scoring categories used to assess physical activity, digital screen time, and dietary adherence.

**Table 3:** Scoring tools and categories The questionnaires used are published under a Creative Commons License.

Scoring tool	Low	Moderate	High
Physical activity (PAQ-C Score)	<2.33	2.33-3.66	>3.66
Digital screen time (hours/day)	<2 hours	2-4 hours	>4 hours
Dietary adherence (FFQ percentile)	<50th percentile	50th-74th percentile	≥75th percentile

Multivariate logistic regression identified obesity (BMI ≥ 95th percentile) as the strongest predictor of adolescent hypertension (aOR: 4.22, 95% CI: 2.88-6.19; p < 0.001). Recreational screen time (>3 hours/day) had a stronger association with hypertension (aOR: 3.12, 95% CI: 2.10-4.02) than educational screen time (aOR: 1.75, 95% CI: 1.21-2.44). Low physical activity (PAQ-C score < 2.33) was independently associated with an increased risk of hypertension (aOR: 1.62, 95% CI: 1.04-2.51; p = 0.033). Table 4 shows the multivariate logistic regression results for predictors of hypertension in adolescents, with Wald test statistics included to assess significance.

**Table 4:** Multivariate logistic regression for predictors of hypertension (subanalysis) BMI: body mass index; OR: odds ratio; CI: confidence interval; PAQ-C: Physical Activity Questionnaire for Older Children; hrs/day: hours per day; χ²: chi-square; p: probability value The asterisk symbol (*) indicates statistical significance at p < 0.05. The Wald statistic is used to assess the significance of each predictor in the logistic regression model. It tests the null hypothesis that the coefficient for a given variable is zero (no effect). A larger Wald statistic indicates stronger evidence against the null hypothesis, suggesting that the predictor is significantly associated with the outcome.

Variable	Adjusted OR (95% CI)	Test statistic (χ²)	Wald statistic (Wald)	p-value
Obesity (BMI ≥ 95th percentile)	4.22 (2.88-6.19)	19.87	10.54	<0.001*
Recreational screen time > 3 hrs/day	3.12 (2.10-4.02)	29.92	14.20	<0.001*
Educational screen time > 3 hrs/day	1.75 (1.21-2.44)	4.82	3.04	0.03*
PAQ-C score < 2.33 (low activity)	1.62 (1.04-2.51)	8.32	6.73	0.033*

A separate logistic regression model, as shown in Table 5, examining total screen time (without differentiating between recreational and educational use) found that overall screen time of more than three hours per day was significantly associated with hypertension (aOR: 2.58, 95% CI: 1.73-3.86; p < 0.001). In this analysis, obesity and low physical activity remained significant predictors.

**Table 5:** Multivariate logistic regression for predictors of hypertension (overall screen time) Odds ratios (ORs) were adjusted for potential confounding variables, including BMI, screen time, and physical activity levels. Asterisks (*) indicate statistical significance at p < 0.05. The Wald statistic is used to assess the significance of each predictor in the logistic regression model. It tests the null hypothesis that the coefficient for a given variable is zero (no effect). A larger Wald statistic indicates stronger evidence against the null hypothesis, suggesting that the predictor is significantly associated with the outcome (hypertension in this case).

Variable	Adjusted OR (95% CI)	Test statistic (χ²)	Wald statistic (Wald)	p-value
Obesity (BMI ≥ 95th percentile)	4.22 (2.88-6.19)	19.87	10.54	<0.001*
Screen time > 3 hours/day (overall)	2.58 (1.73-3.86)	29.92	14.20	<0.001*
PAQ-C score < 2.33 (low activity)	1.62 (1.04-2.51)	8.32	6.73	0.033*

These findings highlight the strong association between obesity, excessive screen time, and adolescent hypertension. This underscores the need for targeted interventions to address obesity prevention, screen time reduction, and increased physical activity to reduce the risk of hypertension among adolescents. The strong association between obesity and hypertension underscores the importance of primary prevention efforts focusing on weight management through balanced nutrition and increased physical activity. Furthermore, prolonged digital screen exposure - whether for recreation or education - should be monitored and minimized as part of comprehensive strategies to prevent adolescent hypertension.

## Discussion

While our study demonstrated significant associations between obesity, screen time, and hypertension, it is important to acknowledge that this is an observational study, meaning that it assesses associations rather than causality. Given the nature of the study design, reverse causality cannot be ruled out. For example, it is possible that adolescents with pre-existing hypertension may engage in more screen time due to lifestyle adaptations rather than directly contributing to hypertension. Future longitudinal and interventional studies are required to delineate these causal pathways better.

The relationship between obesity and hypertension in adolescents has been well-documented in previous studies. Flynn et al. reported that obese adolescents exhibit higher sympathetic nervous system activation and insulin resistance, contributing to hypertension [5]. A meta-analysis by Yang et al. further demonstrated that obese adolescents have an increased lifetime risk of cardiovascular disease owing to persistently elevated BP [15]. Similarly, a longitudinal study by Lande et al. found that obesity accelerates vascular dysfunction and arterial stiffness, predisposing adolescents to develop hypertension [16]. Our findings reinforce these conclusions and highlight the need for early detection and lifestyle modification.

Our study suggests that the combined effects of obesity and excessive screen time may be greater than the sum of their individual effects. Furthermore, the adjusted odds ratio (aOR) for the combined risk group (aOR = 5.14, 95% CI: 3.28-7.56) exceeded the individual odds ratios for obesity (aOR = 4.22) and screen time (aOR = 2.58), suggesting a possible interaction between the effects. However, it is important to note that formal interaction analyses were not performed, and future studies should explore whether the relationship is truly additive or synergistic using more advanced statistical modeling.

Digital screen time has emerged as another critical risk factor for hypertension in young populations. Our study found that a daily screen time exceeding three hours per day was significantly associated with elevated BP, even after controlling for BMI. These results are consistent with those of Cheng et al., who reported that prolonged screen exposure impairs autonomic nervous system regulation by contributing to sustained BP elevation [17]. Leary et al. corroborated that excessive screen time disrupts sleep patterns and increases stress hormone release, culminating in hypertension [18]. A systematic review and meta-analysis by Farhangi et al. similarly showed that prolonged screen-watching behavior was associated with increased SBP and increased odds of hypertension [19].

Notably, entertainment-based screen time (e.g., gaming and social media) may be more strongly associated with hypertension than educational screen use. One plausible explanation is that recreational screening activities often involve longer, uninterrupted periods of sitting, which can coincide with snacking or consuming sugary beverages. Additionally, gaming and social media usage can elicit stress responses or reduce sleep quality more substantially than educational screen use, which exacerbates the risk of hypertension. These distinctions underscore the need for interventions that specifically address sedentary activities in adolescents.

Physical inactivity is another significant predictor of hypertension. The WHO recommends at least 60 minutes of moderate-to-vigorous daily physical activity to maintain optimal cardiovascular health in adolescents [20]. However, our results showed that hypertensive adolescents had substantially lower PAQ-C scores, reflecting decreased engagement in physical activity. These findings align with those of Ekelund et al., who demonstrated that low physical activity levels were linked to increased arterial stiffness and elevated BP in children [21]. Similarly, Pearson and Biddle observed that a sedentary lifestyle exacerbates the combined effects of obesity and digital screen exposure on hypertension risk [22]. These findings reinforce the need for school- and community-based interventions to promote regular physical activity and counteract cardiovascular risks associated with excessive screen use.

Given the strong associations found in our study, structured digital wellness programs should be introduced in schools to educate adolescents about healthy screening habits, sleep hygiene, and stress management. Implementing tech-free zones, scheduled recess activities, and parental education on screen time hygiene could significantly reduce excessive digital exposure. Additionally, pediatricians should consider routine BP screening for adolescents with excessive screen time (>3 hours/day) as part of standard pediatric evaluations. Parents can utilize screen-tracking tools and sleep optimization strategies to regulate screen exposure, particularly in the evening hours, when blue light exposure may disrupt circadian rhythms.

Although lifestyle modifications remain the primary approach for adolescent hypertension management, pharmacological treatment is warranted in specific cases. According to the AAP guidelines, medication should be considered for adolescents with stage 2 hypertension, those with end-organ damage (e.g., left ventricular hypertrophy and renal dysfunction), or those who fail to achieve BP control through lifestyle interventions alone. Commonly prescribed antihypertensive medications include angiotensin-converting enzyme inhibitors, calcium channel blockers, and beta-blockers, depending on the underlying comorbidities [17].

Limitations

This study has several limitations that should be considered when interpreting the results. First, the study relied on self-reported screen time and physical activity, which may have introduced recall bias. Participants may have underreported or overreported their screen time or physical activity levels, affecting the accuracy of these measurements. Future research should incorporate objective tracking methods such as screen-time monitoring apps and wearable devices to improve the accuracy and reliability of data collection.

Second, while our study adjusted for several potential confounders, there may still be unmeasured confounders, such as dietary sodium intake, genetic predisposition, and SES, which could influence both screen-time behaviors and hypertension risk. Future studies should ensure more comprehensive data collection to account for these additional factors and to further refine the analysis.

Another limitation is that, while we observed significant associations between obesity, screen time, and hypertension, a prospective cohort design with repeated BP measurements over time would be necessary to assess the long-term effects and causal relationships between these factors.

Finally, the sample in this study was drawn from urban areas, and the findings may not be generalizable to adolescents in rural areas or those from low- and middle-income countries (LMICs), where different lifestyle factors may influence hypertension risk. Future research should explore these populations to improve the generalizability of the findings and to better understand the impact of urbanization, changing dietary patterns, and increasing digital access to adolescent health.

## Conclusions

This study provides strong evidence that obesity and excessive digital screen time are independent risk factors for early-onset hypertension in adolescents. Our findings indicate that recreational screen time (>3 hours/day) was more strongly associated with hypertension than educational screen time. Public health interventions should focus on limiting nonessential recreational screen time while allowing necessary educational screen use to support learning. Moreover, the combined effect of obesity and screen time on hypertension risk suggests that addressing both factors concurrently may yield the greatest benefits in hypertension prevention. Future longitudinal research should examine whether this relationship is additive or synergistic using advanced statistical methods. To mitigate these risks, we recommend integrating screen time assessments into routine pediatric evaluations. Government-backed digital health literacy campaigns should be launched to promote healthier screen habits among adolescents. Additionally, a multi-tiered intervention approach is needed, incorporating efforts from schools, families, and policymakers to effectively reduce adolescent hypertension prevalence.

By addressing both obesity and excessive screen time through structured interventions, digital wellness initiatives, and routine screening, we can work toward reducing the burden of hypertension-related cardiovascular complications in adolescents.

## Appendices

Appendix A

**Table 6:** PAQ-C Questionnaire Credit: Published under a Creative Commons International License Reference: [10] PAQ-C: Physical Activity Questionnaire for Older Children

PAQ-C Questionnaire is Physical Activity Questionnaire for Older Children (PAQ-C) Name: .............................................. Gender: Male ............ Female .................. Age: ............ Grade Level: ............ School Name: .................................... This questionnaire assesses THE Level of physical activity during the last 7 days. It includes different types of physical activities, such as sports, running, jumping, and recreational activities, dat make you breathe harder and move your body energetically. Instructions: their are no right or wrong answers - this is not a test.		
Question	Response Options	Details
1. During the last 7 days, what physical activities have you done in your free time?		Write how many times you participated in each activity. If none, write "0".
Activity: Jump Rope	1-2 Times	
	3-4 Times	
	5-6 Times	
	7+ Times	
Activity: Skating	1-2 Times	
	3-4 Times	
	5-6 Times	
	7+ Times	
Activity: Soccer	1-2 Times	
	3-4 Times	
	5-6 Times	
	7+ Times	
Activity: Running	1-2 Times	
	3-4 Times	
	5-6 Times	
	7+ Times	
Activity: Riding a Bicycle	1-2 Times	
	3-4 Times	
	5-6 Times	
	7+ Times	
Activity: Swimming	1-2 Times	
	3-4 Times	
	5-6 Times	
	7+ Times	
Activity: Dancing	1-2 Times	
	3-4 Times	
	5-6 Times	
	7+ Times	
Activity: Karate/Martial Arts	1-2 Times	
	3-4 Times	
	5-6 Times	
	7+ Times	
2. How many times in the last 7 days have you participated in physical activities in PE class (e.g., running, jumping, playing sports)?	Never	
	Once	
	Sometimes	
	Often	
	Always	
3. During the last 7 days, what did you do most of the time during recess?	Sat and talked/read	
	Walked around	
	Played lightly	
	Played moderate sports	
	Played actively and ran a lot	
4. During the last 7 days, what did you usually do at lunchtime?	Sat and talked/read	
	Walked around	
	Played lightly	
	Played moderate sports	
	Played actively and ran a lot	
5. After school in the last 7 days, how often did you do physical activities (e.g., sports, dancing, running)?	Never	
	Once per week	
	2-3 times per week	
	4 times per week	
	5+ times per week	
6. In the last 7 days, how many evenings did you do physical activities like running, playing sports, or exercising?	Never	
	Once per week	
	2-3 times per week	
	4-5 times per week	
	6+ times per week	
7. On the last weekend, how often did you do physical activities like running, playing, or exercising?	Never	
	Once per week	
	2-3 times per week	
	4-5 times per week	
	6+ times per week	
8. Over the last 7 days, what best describes your physical activity levels?	Did little or no physical activity	
	Did 1-2 physical activities	
	Did 3-4 physical activities	
	Did 5-6 physical activities	
	Did 7 or more physical activities	
9. During the last 7 days, how often did you play sports or do physical activities like running or dancing?	Never	
	Once per week	
	2-3 times per week	
	4-5 times per week	
	6+ times per week	
10. Were you sick or injured last week, preventing you from doing physical activities?	Yes	If Yes, explain: ..............................................
	No	
Days of the week you were most active:	Monday	
	Tuesday	
	Wednesday	
	Thursday	
	Friday	
	Saturday	
	Sunday	

Appendix B

**Table 7:** Digital screen-time questionnaire Credit: Published under a Creative Commons International License Reference: [12]

Digital Screen-time Questionnaire For the following set of questions, primary activity is defined as the main activity you are engaged in rather than using a television/other screen in the background while performing another activity such as cooking or exercising. Screen use on an average weekday Thinking of an average weekday (from when you wake up until you go to sleep), how much time do you spend using each of the following types of screen as the primary activity? You must answer both hours and minutes. If zero please type "0" in the box.			
Question	Screen Type	Hours	Minutes
Screen use on an average weekday	Television		
	TV-connected devices (e.g. streaming devices, video game consoles)		
	Laptop/computer		
	Smartphone		
	Tablet		
Screen use on an average weeknight	Television		
	TV-connected devices (e.g. streaming devices, video game consoles)		
	Laptop/computer		
	Smartphone		
	Tablet		
Screen use on an average weekend day	Television		
	TV-connected devices (e.g. streaming devices, video game consoles)		
	Laptop/computer		
	Smartphone		
	Tablet		
Background screen use on a regular weekday	Background screen use during the whole day (Monday through Friday)		
Background screen use on a regular weeknight	Background screen use during the evening (Monday through Friday)		
Background screen use on a regular weekend day	Background screen use during the whole day (Saturday or Sunday)		

Appendix C

**Table 8:** Food Frequency Questionnaire (FFQ) This questionnaire is the authors' original creation. Credits: Dr. Sadaf Yasin, Dr. Husnain, and Dr. Fahad Raja Khan

Semi-Quantitative Food Frequency Questionnaire (FFQ) Adapted to Culturally Relevant Dietary Patterns in Pakistan Section 1: Demographic Information Name: ________________ Age: ________________ Gender: ☐ Male ☐ Female ☐ Other Weight (kg): ________________ Height (cm): ________________ Do you follow any specific diet? ☐ No ☐ Vegetarian ☐ Vegan ☐ High-protein diet ☐ Low-fat diet ☐ Other (please specify): ________________ Section 2: Food Consumption Frequency Instructions: For each food item listed below, please indicate how frequently you consume it. Also, provide the usual portion size (small, medium, or large).									
Section	Question	Food Group	Food Item	Never	Rarely (1-2 times/month)	Occasionally (1-2 times/week)	Frequently (3-5 times/week)	Daily	Portion Size (Small/Medium/Large)
Section 1: Demographic Information	Name								
	Age								
	Gender		☐ Male ☐ Female ☐ Other						
	Weight (kg)								
	Height (cm)								
	Do you follow any specific diet?		☐ No ☐ Vegetarian ☐ Vegan ☐ High-protein diet ☐ Low-fat diet ☐ Other						
Section 2: Food Consumption Frequency	Cereals & Grains	Roti (Wheat/Chapati)	☐ ☐ ☐ ☐ ☐	☐ S ☐ M ☐ L					
		Paratha	☐ ☐ ☐ ☐ ☐	☐ S ☐ M ☐ L					
		Rice (Plain/Boiled)	☐ ☐ ☐ ☐ ☐	☐ S ☐ M ☐ L					
Dairy Products	Milk (Cow/Buffalo)	☐ ☐ ☐ ☐ ☐	☐ S ☐ M ☐ L						
		Yogurt (Dahi)	☐ ☐ ☐ ☐ ☐	☐ S ☐ M ☐ L					
		Lassi (Sweet/Salted)	☐ ☐ ☐ ☐ ☐	☐ S ☐ M ☐ L					
Meat & Poultry	Chicken (Curry/Grilled)	☐ ☐ ☐ ☐ ☐	☐ S ☐ M ☐ L						
		Beef (Kebab/Korma)	☐ ☐ ☐ ☐ ☐	☐ S ☐ M ☐ L					
		Mutton (Curry/Grilled)	☐ ☐ ☐ ☐ ☐	☐ S ☐ M ☐ L					
Fish & Seafood	Fish (Fried/Grilled)	☐ ☐ ☐ ☐ ☐	☐ S ☐ M ☐ L						
Legumes & Pulses	Dal (Lentils)	☐ ☐ ☐ ☐ ☐	☐ S ☐ M ☐ L						
		Chana (Chickpeas)	☐ ☐ ☐ ☐ ☐	☐ S ☐ M ☐ L					
Vegetables	Saag (Mustard Greens)	☐ ☐ ☐ ☐ ☐	☐ S ☐ M ☐ L						
		Bhindi (Okra)	☐ ☐ ☐ ☐ ☐	☐ S ☐ M ☐ L					
		Aloo (Potato)	☐ ☐ ☐ ☐ ☐	☐ S ☐ M ☐ L					
Fruits	Mango	☐ ☐ ☐ ☐ ☐	☐ S ☐ M ☐ L						
		Banana	☐ ☐ ☐ ☐ ☐	☐ S ☐ M ☐ L					
		Apple	☐ ☐ ☐ ☐ ☐	☐ S ☐ M ☐ L					
Beverages	Chai (Tea with Milk)	☐ ☐ ☐ ☐ ☐	☐ S ☐ M ☐ L						
		Green Tea	☐ ☐ ☐ ☐ ☐	☐ S ☐ M ☐ L					
		Soft Drinks (Cola/Soda)	☐ ☐ ☐ ☐ ☐	☐ S ☐ M ☐ L					
Snacks & Fast Food	Samosa	☐ ☐ ☐ ☐ ☐	☐ S ☐ M ☐ L						
		Pakora	☐ ☐ ☐ ☐ ☐	☐ S ☐ M ☐ L					
		Biryani	☐ ☐ ☐ ☐ ☐	☐ S ☐ M ☐ L					
Sweets & Desserts	Kheer	☐ ☐ ☐ ☐ ☐	☐ S ☐ M ☐ L						
		Gulab Jamun	☐ ☐ ☐ ☐ ☐	☐ S ☐ M ☐ L					
		Halwa	☐ ☐ ☐ ☐ ☐	☐ S ☐ M ☐ L					
Section 3: Additional Dietary Information	Do you usually eat home-cooked meals or outside food?	☐ Mostly home-cooked ☐ Equal mix of home and restaurant food ☐ Mostly restaurant food							
	How often do you eat fried foods?	☐ Daily ☐ 3-5 times a week ☐ 1-2 times a week ☐ Rarely							
	How often do you eat processed/junk food?	☐ Daily ☐ 3-5 times a week ☐ 1-2 times a week ☐ Rarely							
	Do you take dietary supplements?	☐ No ☐ Yes (Specify): ________________							

## References

[1] Flynn JT, Falkner BE (2011). Obesity hypertension in adolescents: epidemiology, evaluation, and management. J Clin Hypertens (Greenwich).

[2] Jeong SI, Kim SH (2024). Obesity and hypertension in children and adolescents. Clin Hypertens.

[3] Kelly AS, Armstrong SC, Michalsky MP, Fox CK (2024). Obesity in adolescents: a review. JAMA.

[4] Piercy KL, Troiano RP, Ballard RM (2018). The Physical Activity Guidelines for Americans. JAMA.

[5] Flynn JT, Kaelber DC, Baker-Smith CM (2017). Clinical practice guideline for screening and management of high blood pressure in children and adolescents. Pediatrics.

[6] Sorof J, Daniels S (2002). Obesity hypertension in children: a problem of epidemic proportions. Hypertension.

[7] Falkner B, Gidding SS, Portman R, Rosner B (2008). Blood pressure variability and classification of prehypertension and hypertension in adolescence. Pediatrics.

[8] Siddiqui F, Khan M, Alam R (2021). Prevalence and determinants of overweight and obesity among adolescents in Pakistan. BMC Public Health.

[9] (2022). CDC growth charts: United States. CDC growth charts: United States.

[10] Kowalski KC, Crocker PRE, Donen RM (e13237). The Physical Activity Questionnaire for Older Children (PAQ-C) and
Adolescents (PAQ-A) Manual. PeerJ.

[11] (2020). World Health Organization. WHO guidelines on physical activity and sedentary behaviour. https://www.who.int/publications/i/item/9789240015128.

[12] Vizcaino M, Buman M, DesRoches CT, Wharton C (2019). Reliability of a new measure to assess modern screen time in adults. BMC Public Health.

[13] Willett WC (1998). Nutritional Epidemiology. 2nd Ed.

[14] Kenneth Chui KH, Ravi S, Vanzan H (2018). Validation of a semi-quantitative food frequency questionnaire for use WIF an adult South Indian population. Indian J Nutri.

[15] Yang L, Magnussen CG, Yang L (2020). Elevated blood pressure in childhood or adolescence and cardiovascular outcomes in adulthood: a systematic review. Hypertension.

[16] Lande MB, Batisky DL, Kupferman JC (2017). Neurocognitive function in children with primary hypertension. J Pediatr.

[17] Cheng X, Guo Q, Ju L (2024). Association between sedentary behavior, screen time and metabolic syndrome among Chinese children and adolescents. BMC Public Health.

[18] Leary SD, Ness AR, Smith GD, Mattocks C, Deere K, Blair SN, Riddoch C (2008). Physical activity and blood pressure in childhood: findings from a population-based study. Hypertension.

[19] Farhangi MA, Fathi Azar E, Manzouri A, Rashnoo F, Shakarami A (2023). Prolonged screen watching behavior is associated with high blood pressure among children and adolescents: a systematic review and dose-response meta-analysis. J Health Popul Nutr.

[20] (2020). World Health Organization. Guidelines on physical activity and sedentary behaviour. https://www.who.int/publications/i/item/9789240015128.

[21] Ekelund U, Luan J, Sherar LB, Esliger DW, Griew P, Cooper A (2012). Moderate to vigorous physical activity and sedentary time and cardiometabolic risk factors in children and adolescents. JAMA.

[22] Pearson N, Biddle SJ (2011). Sedentary behavior and dietary intake in children, adolescents, and adults. A systematic review. Am J Prev Med.

